# Is There Association between Altered Adrenergic System Activity and Microvascular Endothelial Dysfunction Induced by a 7-Day High Salt Intake in Young Healthy Individuals

**DOI:** 10.3390/nu13051731

**Published:** 2021-05-20

**Authors:** Ana Stupin, Ines Drenjančević, Petar Šušnjara, Željko Debeljak, Nikolina Kolobarić, Ivana Jukić, Zrinka Mihaljević, Goran Martinović, Kristina Selthofer-Relatić

**Affiliations:** 1 Department of Physiology and Immunology, Faculty of Medicine, Josip Juraj Strossmayer University of Osijek, J. Huttlera 4, HR-31000 Osijek, Croatia; ines.drenjancevic@mefos.hr (I.D.); psusnjara1@gmail.com (P.Š.); nikolina.bilic.dujmusic@gmail.com (N.K.); ivana.jukic@mefos.hr (I.J.); zrinka.mihaljevich@gmail.com (Z.M.); 2 Scientific Center of Excellence for Personalized Health Care, Josip Juraj Strossmayer University of Osijek, Trg Svetog Trojstva 3, HR-31000 Osijek, Croatia; selthofer.relatic@gmail.com; 3 Department of Pathophysiology, Physiology and Immunology, Faculty of Dental Medicine and Health, Josip Juraj Strossmayer University of Osijek, Cara Hadrijana 10E, HR-31000 Osijek, Croatia; 4 Clinical Institute of Laboratory Diagnostics, Osijek University Hospital, J. Huttlera 4, HR-31000 Osijek, Croatia; zeljko.debeljak@gmail.com; 5 Department of Pharmacology, Faculty of Medicine, Josip Juraj Strossmayer University of Osijek, J. Huttlera 4, HR-31000 Osijek, Croatia; 6 Department of Software Engineering, Faculty of Electrical Engineering, Computer Science and Information Technology, Josip Juraj Strossmayer University of Osijek, Kneza Trpimira 2B, HR-31000 Osijek, Croatia; goran.martinovic@ferit.hr; 7 Department for Cardiovascular Disease, Osijek University Hospital, J. Huttlera 4, HR-31000 Osijek, Croatia; 8 Department for Internal Medicine, Faculty of Medicine, Josip Juraj Strossmayer University of Osijek, J. Huttlera 4, HR-31000 Osijek, Croatia

**Keywords:** high-salt diet, autonomic nervous system, sympathetic nervous system, heart rate variability, catecholamine, endothelium

## Abstract

This study aimed to test the effect of a 7-day high-salt (HS) diet on autonomic nervous system (ANS) activity in young healthy individuals and modulation of ANS on microvascular endothelial function impairment. 47 young healthy individuals took 7-day low-salt (LS) diet (3.5 g salt/day) followed by 7-day high-salt (HS) diet (~14.7 g salt/day). ANS activity was assessed by 24-h urine catecholamine excretion and 5-min heart rate variability (HRV). Skin post-occlusive reactive hyperemia (PORH) and acetylcholine-induced dilation (AChID) were assessed by laser Doppler flowmetry (LDF). Separately, mental stress test (MST) at LS and HS condition was conducted, followed by immediate measurement of plasma metanephrines’ level, 5-min HRV and LDF microvascular reactivity. Noradrenaline, metanephrine and normetanephrine level, low-frequency (LF) HRV and PORH and AChID significantly decreased following HS compared to LS. MST at HS condition tended to increase HRV LF/HF ratio. Spectral analysis of PORH signal, and AChID measurement showed that MST did not significantly affect impaired endothelium-dependent vasodilation due to HS loading. In this case, 7-day HS diet suppressed sympathetic nervous system (SNS) activity, and attenuated microvascular reactivity in salt-resistant normotensive individuals. Suppression of SNS during HS loading represents a physiological response, rather than direct pathophysiological mechanism by which HS diet affects microvascular endothelial function in young healthy individuals.

## 1. Introduction

Excessive dietary salt intake presents an emerging issue in almost all part of the world, including Croatia. Since average daily salt intake in Croatia in 2008 (Salt mapping in Croatia—Croatian action on salt and health (CRASH): average 11.6 g of salt/day; 13.3 g/day for men and 10.2 g/day for women) was more than twice the recommended daily intake according to the World health Organization (WHO) (5 g of salt/day) [1], Croatian Institute of Public Health and the Ministry of Health developed the Strategic Plan for Reduction of Salt Intake aiming to reduce daily salt intake by 4% each year, from 11.6 g in 2015 to 9.3 g in 2019 [2]. So far, only preliminary data on daily salt intake in Croatia from 2019 are available, which show that average daily salt intake has decreased for 1.6 g of salt per day in a last 12 years (1.9 g of salt/day in men and 1.0 g of salt per day in women) and also indicate that there is a need for continuation of efforts to bring the salt intake in Croatia closer to the values recommended by the WHO [3].

Increased dietary salt intake, increased activation of the renin-angiotensin system (RAS) and elevated sympathetic nervous system (SNS) activity has been leading risk factors and contributors for the development and progression of arterial hypertension [4,5,6,7,8,9]. Importantly, in recent years it became evident that high-salt (HS) dietary intake may affect vascular function even in the absence of changes in arterial blood pressure (BP) [4,9]. Moreover, the development of vascular endothelial dysfunction which is characterized by altered endothelium-dependent vasodilation, as well as by systemic endothelial cells’ activation and endothelial-leukocyte interaction, is now considered as one of the earliest adverse effects of the HS loading on the cardiovascular (CV) system [9]. For example, the short-term HS diet (7-day HS diet) impairs endothelium-dependent vasodilation in both macro- (flow-mediated dilation of the brachial artery) and microcirculation (microvascular response to vascular occlusion, local heating and administration of acetylcholine (ACh)) in healthy individuals independently of BP changes, or changes in body composition and body fluid status [10,11,12,13,14,15,16]. Thus, the concept of salt-sensitivity cannot be limited only to the effect of dietary salt modulation on BP, but obviously to its effect on vascular function and on the CV system in general as well [17].

We have earlier reported that in normotensive salt-resistant individuals’ microvascular reactivity in response to vascular occlusion following 7-day HS diet was strongly predicted by both antioxidant capacity and the level of the RAS activity, rather than antioxidant capacity alone [18]. These results potentiated the key role of normal RAS function in maintaining vascular homeostasis and indicated that the alteration in RAS during dietary salt perturbation could be involved in concomitant changes in endothelial function [18]. Since the SNS activity presents a significant regulator of the RAS activation, it became evident that in order to clarify the pathogenic relation between HS diet and impaired vascular function, it is necessary to investigate the effect of salt loading on major regulating systems, including RAS and autonomic nervous system (ANS) activity, in otherwise healthy individuals.

In physiological conditions, salt intake is inversely related to RAS activation, which is also modulated by concomitant changes in the SNS activity due to salt intake [19,20]. For example, plasma and urine norepinephrine and epinephrine levels are shown to be decreased in normotensive individuals on a HS diet, but the salt-induced suppression of the SNS activity was not observed in salt-sensitive hypertensive patients [21]. Suggested mechanisms that mediate interaction between salt intake and SNS activity are rather complex, indicating (1) that high dietary salt intake can induce a reflex reduction of the SNS activity by cardiopulmonary receptors activation in response to increased plasma volume; (2) acute increase of sodium concentration and osmolality in both plasma and cerebrospinal fluid can induce an increase of SNS activity [22,23]; (3) renal SNS activity acts as a moderator in the RAS response to changes in salt load (activation of β1 adrenoceptors placed in renin-producing JGE cells stimulates renin release) [24,25], but also (4) that the local brain RAS has ability to modulate SNS activity [26]. Altogether these results support the hypothesis of potentially altered feedback loop between RAS suppression due to salt loading and changed SNS activity, which may consequently affect vascular function, a major effector in maintaining total peripheral resistance and BP. Even though numerous studies in experimental animals have unequivocally shown a key role of RAS suppression in the development of endothelial dysfunction in HS intake [9,27], and studies in healthy individuals increasingly confirm these findings [18,28], the potential role of the ANS modulation during HS loading on consequent changes of vascular endothelial function has not been clarified yet.

Thus, the aim of the present study was to test the effect of a 7-day HS diet on ANS activity in young healthy individuals by assessing 24-h urine catecholamine excretion and 5-min heart rate variability (HRV), and to examine whether the modulation of the SNS activity (by mental stress test) following 7-day HS diet would affect microvascular endothelial function impairment caused by HS diet in otherwise healthy normotensive individuals.

## 2. Materials and Methods

### 2.1. Study Population

Forty-seven young healthy individuals (19 women and 28 men) age ranged from 18–29 years took part in the present study. Subjects were recruited by the advertisement at the Faculty of Medicine Josip Juraj Strossmayer University of Osijek. Exclusion criteria for participation in the present study were as follows: obesity [body mass index (BMI) > 30 kg/m^2^], hyperlipidemia, hypertension, diabetes, renal impairment, coronary artery, cerebrovascular and peripheral artery disease, just as taking oral contraceptives, or any other drugs/agents that could affect the endothelium. In order to exclude the potential effect of sex hormones fluctuation during menstrual cycle on endothelial function, women were assigned to the study protocol in different phases of the menstrual cycle (randomized). Each subject provided written informed consent. All protocols and procedures used in the present study were in accordance with the standards set by the latest revision of the Declaration of Helsinki and were approved by the Ethical Committee of the Faculty of Medicine, University of Osijek (Cl: 602-04/15-08/08; No: 2158-61-07-15-68). The present study is a part of registered clinical trial on the effect of HS diet on microvascular function in healthy young individuals at ClinicalTrials.gov (ID NCT02727426 Dietary Salt and Microvascular Function), which is extended to additional outcome related to the non-invasive assessment of autonomic nervous system activity.

### 2.2. Study Protocol

The present study was designed as a controlled clinical experiment, in which all subjects during first 7 days were instructed to maintain a low-salt (LS) diet according to the DASH eating plan aimed to achieve a salt intake of 3.75 g of salt per day (DASH eating plan; US Department of Health and Human Services, 2006). LS diet, considered as “wash-out” period, was followed by 7 days HS diet, which implied intake of approximately 14.7 g of salt per day—3.75 g of salt per day in diet (DASH diet) supplemented with additional 11.2 g of salt per day in a form of a salt powder (commercially available iodinated kitchen sea salt). All measurements were made after 7-day LS diet (considered a “wash-out” period) (LS condition) and repeated after 7-day HS diet (HS condition.).

The study was conducted in two stages. At stage 1 (23 subjects; 7 women and 16 men) subjects underwent a functional and biochemical assessment of the activity of the ANS system before (LS) and after a HS diet (HS). Functional assessment included a 5-min measurement of heart rate variability (HRV), and biochemical assessment included the determination of catecholamine values in 24-h urine samples. The following measurements were also performed: arterial blood pressure (BP) and heart rate (HR) measurement, assessment of anthropometric parameters, assessment of adherence to a dietary protocol based on 24-h urine sodium excretion, venous blood sampling for standard biochemical measurements and functional assessment of microvascular endothelial function by laser Doppler flowmetry (LDF).

**Figure 1:**
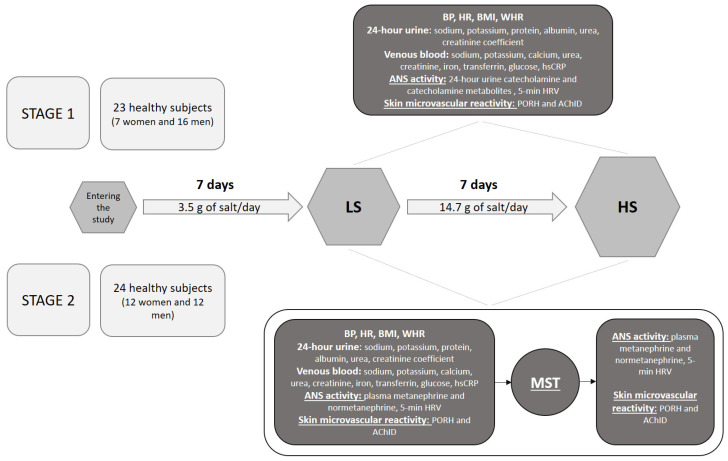
Study timeline and protocol. LS—low salt; HS—high-salt; BP—blood pressure; HR—heart rate; BMI—body mass index; WHR—waist-to-hip ratio; hsCRP—high sensitivity C reactive protein; ANS—autonomic nervous system; HRV—heart rate variability; PORH—post-occlusive reactive hyperemia; AChID—acetylcholine-induced dilation; MST—mental stress test.

According to the results of the stage 1, the stage 2 was designed to evaluate whether potential acute modulation of the SNS system will affect microvascular endothelial responses to dietary salt perturbation (24 subjects: 12 women and 12 men). Thus, at this stage, at LS and HS study visit, beside abovementioned measurement, subjects underwent acute stimulation of the SNS activity in the form of a mental stress test (MST). The effect of the acute ANS modulation on microvascular endothelial function was assessed by performing measurement of 5-min HRV, venous blood sampling (for plasma metanephrines’ assessment) and functional microvascular measurements immediately before and after the MST at LS and HS study visits. Protocol of the study is presented in Figure 1.

All the measurements were performed in the morning after an overnight fasting. Study was performed in the Laboratory for Clinical and Sport Physiology, Department of Physiology and Immunology at Faculty of Medicine Josip Juraj Strossmayer University of Osijek, Osijek, Croatia.

### 2.3. Hemodynamic and Anthropometric Measurement

At each study visit, after a 15 min rest in a seated position, arterial BP and heart rate (HR) were measured using an automated oscillometric sphygmomanometer (OMRON M3, OMRON Healthcare Inc., Osaka, Japan). The average of three consecutive measurements was taken as the final values of BP and HR.

Subjects’ height (m) and weight (kg) were measured to calculate body mass index (BMI). In addition, waist and hip circumference was measured to calculate waist-to-hip ratio (WHR).

### 2.4. 24-h Urine Samples Analysis

Urine was collected during the last 24-h of the LS and HS conditions according to the given instructions. 24-h urine samples were analyzed for sodium, potassium, creatinine coefficient, albumin, protein and urea concentration. Daily salt intake based on 24-h urinary sodium excretion was calculated using appropriate formula [1-g salt (NaCl) = 393.4 mg Na = 17.1 mmol Na]. 24-h urine was also analyzed for dopamine, noradrenaline, adrenaline, homovanillic acid, normetanephrine, metanephrine and vanillylmandelic acid using commercially available kits (Recipe, Munich, Germany) and Nexera HPLC instrument (Shimadzu, Kyoto, Japan) equipped with electrochemical detector (Recipe, Munich, Germany). 24-h urine samples analysis was performed at the Clinical Institute of Laboratory Diagnostics, University Hospital Osijek, Osijek, Croatia.

### 2.5. Venous Blood Samples Analysis

A venous blood sample was taken at each visit, and analyzed for plasma electrolytes (sodium, potassium, calcium), urea, creatinine, iron, transferrin, high sensitivity C reactive protein (hsCRP) and fasting blood glucose using standard laboratory methods.

In subjects who underwent an MST, blood sampling for metanephrines’ determination was performed at the beginning of the visit (as noted above) and repeated immediately after the MST. Plasma metanephrine and normetanephrine level was measured by commercially available kit from Recipe (Munich, Germany) and LC-MS/MS instrument Nexera with LCMS-8050 detector from Shimadzu (Kyoto, Japan). Venous blood samples analysis was performed at the Clinical Institute of Laboratory Diagnostics, University Hospital Osijek, Osijek, Croatia.

### 2.6. Functional Assessment of Skin Microvascular Endothelial Function

Laser Doppler flowmetry (LDF) (MoorVMS-LDF, Axminster, UK) was used to assess microvascular endothelial function by induction of post-occlusive reactive hyperemia (PORH), and iontophoretic administration of acetylcholine (endothelium-dependent vasodilation). The procedures for the LDF measurements (PORH and AChID) are described in detail in earlier papers of our study group [18,29,30]. LDF measurements were performed at LS and HS study visit, and at the stage 2 of the study, LDF measurement was repeated at LS and HS study visit immediately after the MST, using the same protocol.

The PORH test was performed by microvascular blood flow measurement before, during, and after the release of 1-min vascular occlusion. Blood flow rate was determined by original software calculating the area under the curve (AUC) during baseline flow, occlusion, and reperfusion. The difference in percentage of flow change during reperfusion and occlusion in relation to baseline (R-O% increase) was expressed as the final result. Spectral analysis of PORH LDF signal was performed using original MoorVMS-PC v4.0 software. This software measures the power spectral density of LDF signal, and the final result was the percentage of the sum of power signal values in frequency bands related to endothelial activity (0.008–0.02 Hz), sympathetic activity (0.02–0.05 Hz), myogenic activity (0.05–0.15 Hz), respiratory activity (0.15–0.6 Hz) and cardiac activity (0.6–2.0 Hz).

Iontophoretic ACh administration was done following 5 min baseline recording by iontophoresis of positively charged vasodilator ACh (1%) using anodal current (seven 0.1 mA direct electric current pulses for 30 s with 30 s between each pulse). Blood flow rate in this test was also determined by software calculating the AUC during baseline flow and ACh administration, and the blood flow increase following ACh administration in relation to baseline flow was taken as the final result (ACh blood flow increase).

### 2.7. Measurement of 5-min Heart Rate Variability

Five-minute heart rate variability (HRV) was measured using a Heart Rhythm Scanner PE Limited Edition with implemented USB ECG recorder (Biocom Technologies, Poulsbo, WA, USA). In this case, 5-min HRV measurements were acquired at LS and HS study visit, in a resting supine position, and in a quiet, temperature-controlled room. At the stage 2 of the study, 5-min HRV measurement was repeated immediately after the MST, using the same protocol.

The ECG electrodes were placed on the arms (above the wrists) of the subjects, and recording began when the device confirmed a sufficiently high-quality ECG record. The HRV device has implemented automatic detection of abnormal heartbeat (artifacts) and also gives the possibility of correcting artifacts manually. Original software provided by the manufacturer was used to perform autonomic balance test which included time domain and frequency domain analysis of recorded 5-min ECG sessions.

Time-domain indices of HRV quantify the amount of variability in measurements in the time between (normal) successive heartbeats. Time domain analysis provides automatic calculation of the following parameters: Mean HR (beats per minute), Mean RR (ms), SDNN (standard deviation of normal-to-normal (NN) intervals) (ms), RMS-SD (the root mean square of successive differences between normal heartbeats) (ms) and pNN50 (the percentage of adjacent NN intervals that differ from each other by more than 50 ms) (%).

Frequency-domain analysis uses Fast Fourier Transformation (FFT) to separate HRV into its component rhythms that operate within different frequency ranges, and provides the distribution of absolute power (TP; the signal energy found within a frequency band) (ms^2^/Hz) into three frequency bands; VLF (very-low frequency) power spectrum evaluated in the range from 0.0033 to 0.04 Hz, LF (low-frequency) power spectrum (parasympathetic and sympathetic activation) evaluated in the range from 0.04 to 0.15 Hz and HF (high-frequency) power spectrum (parasympathetic activation) evaluated in the range from 0.15 to 0.4 Hz.

### 2.8. Mental Stress Test

At the stage 2 of the study, during LS and HS study visit, MST was used to provoke subject’s SNS activity. MST was chosen as a tool for provoking SNS activity due to its availability, non-invasiveness and not causing significant discomfort in the subjects. Stressor was a 7-min long arithmetic test in form of, at first sight, simple arithmetic operation (e.g., 578 − 174 = 404). Subject’s task was to answer is the operation correct or incorrect with time limit of 5 s for each task. If subject did not offer answer, it was recorded as incorrect. After each answer, subject got feedback information if the answer was correct or not by different sound signals. In a 7-min test subject got approximately 60 different arithmetic tasks, and after the test got information of his success. Immediately after MST participants were subjected to a repeated venous blood sampling (for plasma metanephrines’ analysis), 5-min HRV test and LDF measurement of microvascular endothelial function.

### 2.9. Statistical Analysis

All results are reported as the arithmetic mean ± standard deviation (SD). The normality of data distribution was assessed by the Kolmogorov–Smirnov normality test. Differences between measurements done before and after HS diet protocol (LS vs. HS) were assessed using paired t-test, or the Wilcoxon rank-sum test when variables were not normally distributed. After implementation of MST, the differences between data obtained before and after MST test at LS and HS study visit (LS, LS + MST, HS, HS + MST) were assessed using One Way Repeated Measures ANOVA Test, and Tukey test for post-hoc analysis, as appropriate. Pearson’s or Spearman’s correlation test was used to determine the correlations between microvascular endothelial function (PORH and AChID) and corresponding variables (salt intake, 5-min HRV indices: RMS-SD, VLF, LF, HF, LF/HF; 24-h urine catecholamine and catecholamine metabolites excretion: dopamine, noradrenaline, adrenaline, homovanillic acid, normetanephrine, metanephrine, vanillylmandelic acid). To examine which of the abovementioned variables more significantly predict changes in microvascular function following the HS diet, a multiple linear regression model was used. *p* < 0.05 was considered statistically significant. SigmaPlot, version 11.2 (Systat Software, Inc., Chicago, IL, USA) was used for statistical analysis.

## 3. Results

All participants were lean and normotensive, had normal renal function, serum electrolytes, fasting blood glucose and hsCRP level when entering the study protocol. They all completed 2 consecutive weeks’ dietary salt perturbation.

Anthropometric, hemodynamic and biochemical responses to a 7-day HS diet are presented in Table 1. 7-day HS diet did not induce any significant change in BMI and WHR in young healthy population (Table 1). 7-day HS diet did not induce any significant change in SBP, DBP and MAP values compared to LS diet (Table 1). Moreover, according to change in MAP following HS diet compared to LS conditions (≤5 mmHg), all participants were characterized as salt resistant. Since large increase in salt intake during 7-days was not accompanied with concomitant increase in BP values, all the observed (and furtherly presented) effects of a 7-day HS diet could be characterized as independent of BP. HR was not significantly changed by a 7-day HS diet compared to LS (Table 1). Serum sodium and potassium concentration significantly increased, while calcium and transferrin concentration significantly decreased following 7-day HS diet compared to LS conditions, but still all the values were within normal reference range (Na 137–146 mmol/L, K 3.9–5.1 mmol/L, Ca 2.14–2.53 mmol/L, transferrin 2.00–3.60 g/L) (Table 1), supporting normal modulation of RAS activity with dietary salt intake. There was no significant difference in serum urea, creatinine, iron, fasting glucose and hsCRP levels following HS diet compared to LS (Table 1). There is some discrepancy between predetermined (3.5 g of salt/day) and the actual salt intake during LS diet (6.4 g of salt/day), which could be attributed to a challenging LS diet goal. Still, as expected, 24-h urine sodium excretion as well as calculated daily salt intake significantly increased following HS diet compared to LS. 7-day HS diet did not significantly affect 24-h urinary total volume, creatinine coefficient, urea, potassium, protein and albumin excretion compared LS diet (Table 1).

### 3.1. Stage 1 Study Results—The Effects of 7-Day High-Salt (HS) Loading on Autonomic Nervous System (ANS) Activity and Microvascular Endothelial Function

The effect of a 7-day HS diet on 24-h urine catecholamine and catecholamine metabolites excretion is presented in Table 2. 7-day HS diet induced statistically significant decrease in 24-h urine noradrenaline, metanephrine and normetanephrine excretion compared to LS. Excretion of other measured catecholamine (dopamine and adrenaline) and catecholamine metabolites (homovanillic acid and vanillylmandelic acid) tended to decrease following HS diet (compared to LS) but the difference did not reach the level of statistical significance.

The effect of a 7-day HS diet on 5-min HRV is presented in Table 3. Even though Mean RR, SDNN and RMS-SD tended to decrease following 7-day HS diet compared to LS, changes in 5-min HRV time domain analysis parameters did not reach the level of statistical significance. Regarding 5-min HRV frequency domain analysis indices, LF significantly decreased while HF and LF/HF ratio tended to decrease following HS diet compared to LS.

Seven-day HS diet significantly decreased both PORH (R-O% LS 145.1 ± 42.0 vs. HS 124.7 ± 30.3, *p* = 0.034), and ACh-induced dilation (AChID) (AChID flow increase LS 17.4 ± 4.8 vs. HS 13.9 ± 4.0, *p* = 0.002) of forearm skin microcirculation compared to LS diet.

There was a significant weak negative correlation between salt intake and PORH (r = −0.433, *p* = 0.031), and significant moderate negative correlation between salt intake and AChID (r = −0.620, *p* < 0.001). In addition, there was a significant weak negative correlation between salt intake and 24-h urine metanephrine excretion (r = −0.412, *p* = 0.009). There was no other significant correlation between salt intake and 24-h urine catecholamine excretion, just as there was no significant correlation between salt intake and measured indices of 5-min HRV. Both PORH and AChID significantly correlated only with salt intake, while there was neither significant correlation between PORH or AChID and 24-h urine catecholamine excretion, nor PORH or AChID and measured indices of 5-min HRV.

When using a multiple linear regression model to analyze which of the parameters (salt, RMS-SD, LF, HF, LF/HF, noradrenaline, normetanephrine, metanephrine) most strongly predict the PORH and AChID value, the results showed that only salt intake is a parameter that most strongly predicts the PORH value (r^2^ = 0.791, *p* = 0.018), while salt intake and adrenaline/metanephrine together are strongly negatively associated with AChID (adrenaline r^2^ = 0.707, *p* = 0.001; metanephrine r^2^ = 0.704, *p* = 0.010), rather than salt intake alone (r^2^ = 0.443, *p* = 0.009).

### 3.2. Stage 2 Study Results—The Effects of Increased Sympathetic Nervous System (SNS) Activity (by Mental Stress Test; MST) during 7-Day High-Salt (HS) Loading on Microvascular Endothelial Function

**Figure 2:**
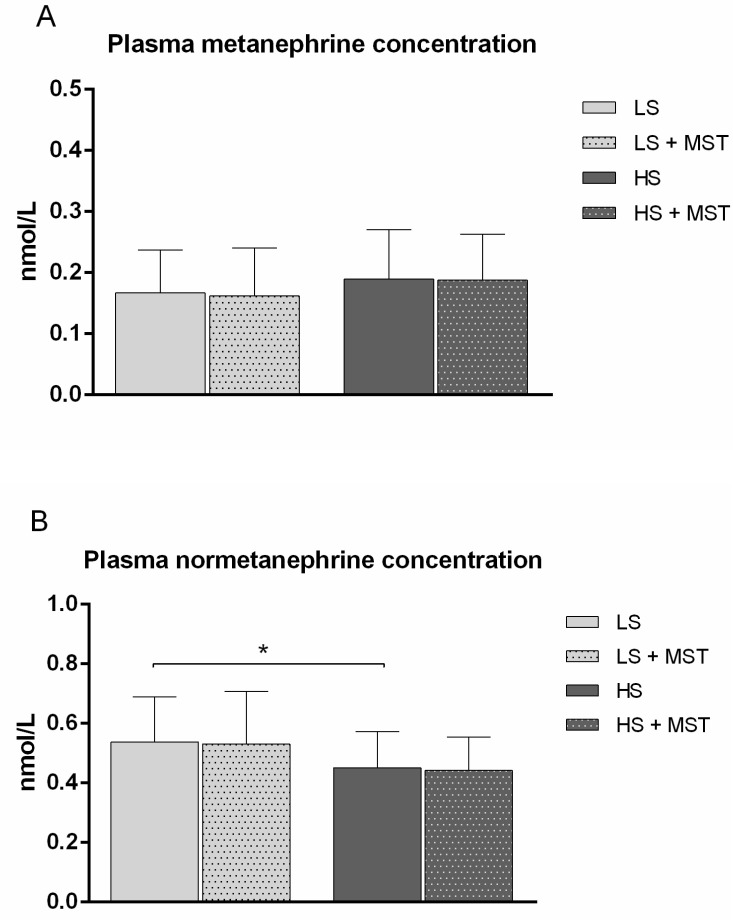
The effect of mental stress test on plasma metanephrine (**A**) and normetanephrine (**B**) concentration at low-salt (LS) and high-salt (HS) diet conditions in young healthy individuals. *N* = 24 (12 women and 12 men). LS—low salt; HS—high salt; MST—mental stress test; *N*— number of subjects. Data are presented as average ± SD. One Way ANOVA Repeated Measures, * *p* < 0.05, LS diet vs. HS diet.

Plasma normetanephrine concentration significantly decreased (Figure 2B), while plasma metanephrine concentration was not significantly changed following 7-day HS diet compared to LS (Figure 2A). MST was not accompanied by significant changes in measured metanephrine and normetanephrine concentration, at both LS and HS diet conditions (Figure 2A,B).

Even though 5-min HRV time-domain and frequency-domain analysis parameters were not significantly changed following 7-day HS diet, similarly with the results from the stage 1 of the present study both LH and HF, as well as LF/HF tended to decrease following HS diet compared to LS, indicating a suppression of both, but more dominantly SNS than parasympathetic nervous system (PNS) activity following HS diet (Table 4). In addition, even though MST at both LS and HS condition did not induce statistically significant change in time-domain and frequency-domain analysis indices, there was a tendency of LF/HF increase following MST at both LS (for 27.2%) and HS (for 11.1%) study visit indicating that MST caused a shift of SNS and PNS balance towards sympathetic dominance (Table 4).

**Figure 3:**
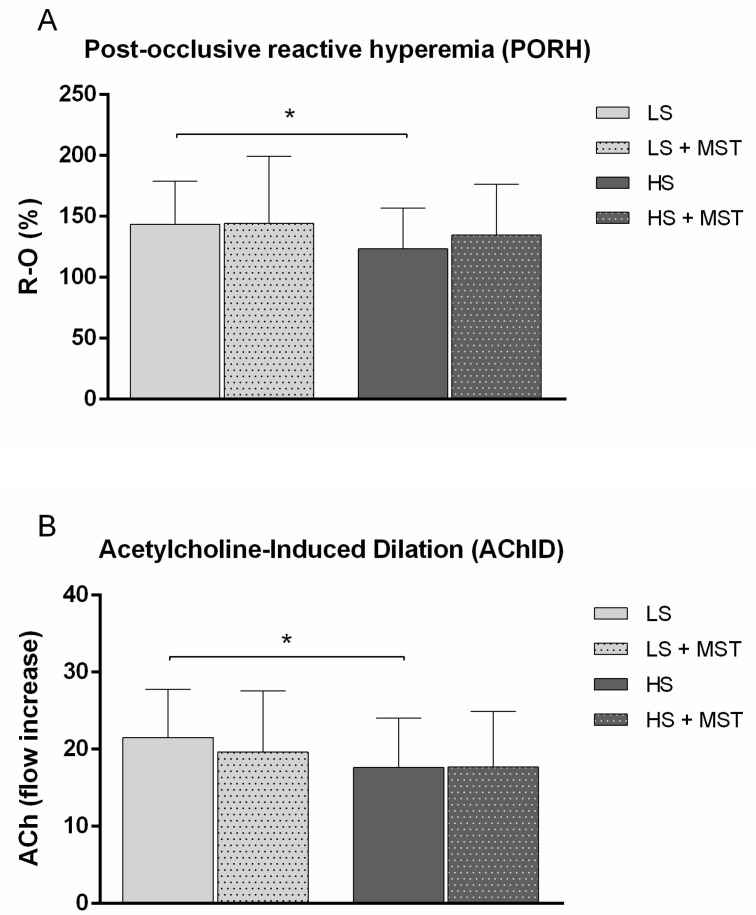
The effect of mental stress test on post-occlusive reactive hyperemia (PORH) (**A**) and acetylcholine-induced dilation (AChID) (**B**) of forearm skin microcirculation measured by laser Doppler flowmetry (LDF) at low-salt (LS) and high-salt (HS) diet conditions in young healthy individuals. *N* = 24 (12 women and 12 men). LS—low salt; HS—high salt; MST—mental stress test; *N*—number of subjects. Data are presented as average ± SD. One Way ANOVA Repeated Measures, * *p* < 0.05, LS diet vs. HS diet.

Consistent with the results from the stage 1 of the present study, PORH (Figure 3A) and AChID (Figure 3B) of forearm skin microcirculation were significantly decreased following 7-day HS compared to LS diet. Interestingly, a tendency of PORH to increase following MST was observed at HS diet condition, but without reaching the level of statistical significance. When the MST-dependent delta PORH changes at LS and HS were compared, this change was not statistically significant (PORH ΔR-O% after vs. before MST at LS 0.59 ± 62.73 vs. HS 8.19 ± 40.35, *p* = 0.591). Moreover, MST did not significantly affect AChID at both LS and HS diet condition (Figure 3A,B). MST-dependent delta AChID changes at LS and HS were not statistically significant (AChID Δ flow increase after vs. before MST at LS—1.84 ± 9.62 vs. HS 0.07 ± 7.63, *p* = 0.450).

In order to examine what is the potential origin of PORH trend to increase following MST, at both LS and HS diet condition, FFT analysis of LDF PORH signal was performed and data are presented at Table 5. Interestingly, FFT of PORH signal showed that MST at both LS and HS diet condition tended to increase the proportion of power signals (% of total signal) in frequencies subintervals related to cardiac and respiratory activity, but not in those related to endothelial and local vascular sympathetic activity (Table 5). Such finding corresponds to unchanged AChID of forearm microcirculation following MST at both LS and HS, which is considered as endothelium-dependent dilation.

## 4. Discussion

This is the first functional in vivo study which investigated the effect of 7-day HS loading on the ANS activity and its potential relationship with impaired microvascular endothelium-dependent reactivity in young healthy individuals. The strength of the present study is its challenging design in 2 stages: we assessed the activity of the ANS, by measuring both functional (5-min HRV) and biochemical (24-h urine catecholamine excretion) biomarkers in relation to dietary salt perturbation (stage 1), and we tested whether these changes in the ANS activity are directly involved in the development of microvascular endothelial dysfunction due to HS loading (stage 2). The novel findings of the stage 1 of this study are that 7-day HS diet significantly reduced the 24-h urine catecholamine excretion and decreased LF 5-min HRV (and LF/HF ratio), indicating that short-term HS loading results in suppressed SNS activity, together with impaired microvascular reactivity. Stage 2 of this study demonstrated that MST at both LS and HS diet condition did not induce significant alteration in microvascular endothelium-dependent responses (unchanged AChID and unchanged endothelial activity component in a spectral analysis of PORH signal), indicating that suppression of SNS during HS loading represents a physiological response associated with suppression of the RAS due to HS intake, rather than direct pathophysiological mechanism by which HS diet affects microvascular endothelial function in young healthy individuals.

### 4.1. High-Salt Diet and Autonomic Nervous System Activity

Available experimental data indicate that HS intake can impact the ANS activity [21], although the extent of this interaction and its potential contribution to changes in endothelial function remain controversial. Several studies in which HS intake was not accompanied by BP changes have reported suppression of the SNS with HS intake [31,32,33]. Conversely, when BP has been found to be salt-sensitive, concurrent activation of the SNS has been shown to occur [21,32,34,35]. Campese et al. reported that HS intake significantly decreased plasma norepinephrine level in normal subjects and in salt-resistant patients but not in salt-sensitive patients [34]. Similarly, muscle sympathetic nerve activity, plasma concentration and urinary excretion of norepinephrine were significantly reduced, whereas plasma concentration of epinephrine was unchanged and urinary excretion of epinephrine was reduced by HS intake in salt-resistant subjects [36]. In the present study we found significantly reduced noradrenaline, normetanephrine and metanephrine 24-h urine level (Table 2), as well as plasma normetanephrine level following 7-day HS diet in young healthy (normotensive) individuals, demonstrating that our participants are salt-resistant (Figure 1B). These results are in line with the results of recent large Cochrane systematic review (185 intervention studies of 12.210 individuals) showing that mean dietary sodium intake reduction from 11.5 g per day to 3.8 g per day significantly increased plasma adrenaline (14%) and noradrenalin (27%) level in both normotensive and hypertensive individuals [37]. Additionally, obtained results are consistent with the results of earlier studies on this issue [21,36], showing that in healthy normotensive individuals HS diet reduced catecholamine (metanephrine) levels in both 24-h urine and serum samples, which is clearly related to suppressed SNS activity following HS loading in healthy individuals.

HRV is commonly used for functional assessment of the ANS activity, influenced by both SNS and PNS (vagal) activity, and reported using time domain and frequency domain analyses [38,39,40]. It has been demonstrated that sodium intake is linked to HRV. For example, in healthy normotensive middle-aged women, HS diet resulted in increased systolic BP, decreased HR and increased HF HRV, indicative of increased cardiac vagal tone [41]. Furthermore, other study showed that a LS diet does not alter HRV in normotensive men and women with asthma [42]. A randomized-controlled trial in normotensive individuals reported that HS intake decreased LF, and increased SDNN, RMSSD and HF compared to LS condition, suggesting that HS loading reduced sympathetic and increased vagal activation [43]. Additionally, Minami et al. showed that the responses of the SNS and PNS (assessed by HRV) to HS intake are attenuated in salt-sensitive vs. salt-resistant essential hypertensive patients [44]. The results of the present study demonstrated that 7-day HS loading significantly decreased LF HRV (produced by both PNS and SNS influences), but also tended to decrease SDNN, RMSSD and HF HRV (reflecting PNS activation), which together resulted in decreased (but not statistically significant) LF/HF ratio (prominent influence of SNS; Table 3). Obtained HRV results are in line with the results of biochemical assessment of SNS activity in the present study, reporting decreased concentrations of noradrenaline, normetanephrine and metanephrine in 24-h urine after HS intake (Table 2). Taken together, present results suggest that HS diet attenuates SNS activity in salt-resistant individuals, while results on PNS activity are not so unambiguous. Thus, clarification of this issue could be of key importance to understand salt-sensitive vascular changes, because in healthy subjects the alterations of autonomic balance could provide prognostic information on CV events, beyond traditionally accepted CV risk factors, such as age, sex, lipid profile, BP level, diabetes etc.

Seven-day HS diet significantly impaired forearm skin microvascular reactivity in response to vascular occlusion (PORH) and iontophoresis of ACh (AChID) in the absence of BP changes in young healthy individuals (in agreement with our and other previous findings), which was discussed in detail in the recent paper of our research group [14,15,18]. The novelty of the present study is that impaired endothelium-dependent (PORH and AChID) microvascular vasodilation was not directly correlated with ANS activity changes (24-h urine catecholamine level or HRV parameters) but only negatively correlated with salt intake. Importantly, despite the fact that skin vasculature is under extensive ANS innervation, the present results suggest that changes of the ANS within normal physiological feedback response do not have such a pronounced effect on the cutaneous microcirculation reactivity, which is a commonly used model for assessment of generalized microvascular function [45]. In addition, multiple linear regression analysis showed that only salt intake (but not parameters related to the ANS activity) strongly predicts microvascular PORH, but interestingly, that salt intake and adrenaline/metanephrine are strongly negatively associated with microvascular AChID, rather than salt intake alone. This negative association between salt intake and adrenaline/metanephrine level with AChID in healthy individuals potentially indicate that eventual increase in the SNS activity (observed in salt-sensitive hypertensive patients) could result in an even more significant deterioration of endothelial function than one observed in salt-resistant individuals, as were in the present study.

### 4.2. High-Salt Diet, Autonomic Nervous System and Microvascular Endothelial (Dys)function

To test whether suppression of the SNS activity has a role in functional impairment of microvascular (endothelium-dependent) reactivity which occurs with HS diet, participants in the stage 2 of the present study were subjected to the MST, which presented a tool for provoking and increasing SNS activity. It is well accepted that acute laboratory maneuvers that evoke mental stress have significant effects on ANS modulation [46]. Among sympathetic stimulation tests (e.g., postural stimulation, insulin tolerance test, cold pressor test), MST is considered as less intense stressful condition [47]. In the present study, MST did not induce significant changes in plasma metanephrine and normetanephrine levels at both LS and HS conditions. This result could be attributed to the fact that during less intense stressful conditions, such as MST, it is expected that metanephrine and normetanephrine responses are proportionally lower than those of catecholamine [48]. However, due to technical demands and challenging procedures we were not able to perform plasma catecholamine concentration measurement, which could be considered as a limitation of the present study.

Interestingly, in the present study MST at both LS and HS conditions tended to shift the autonomic balance toward greater sympathetic activity (increased LF/HF ratio) although there was a difference in the pattern of HRV indices changes following MST at LS and HS condition. Allen et al. also shown that dietary sodium influenced the effect of mental stress on HRV, as diet-by-mental stress interactions were significant for HR, RMS-SD, HF, normalized LF and LF/HF ratio [43]. HRV in the present study was used as a tool to assess ANS activity in regard to HS diet and MST; however, detailed analysis of how dietary salt perturbation together with stress exposure affect HRV remains to be performed in further studies. The fact that increased SNS activity following MST in the present study was not accompanied by a significant increase in plasma metanephrine’s metabolites, but only by a tendency of LF/HF increase, could be attributed to highly challenging research methodology, rather than considered as a potential study limitation.

The focus of the present study was set on testing whether change in the ANS activity due to HS loading, take part in impaired microvascular endothelium-dependent vasodilation in young healthy individuals. At LS diet condition, characterized by increased resting sympathetic activity (compared to HS), microvascular reactivity (both PORH and AChID) was not significantly changed following MST, indicating that modest additional changes in autonomic balance did not have significant effect on microvascular endothelium-dependent responses in normotensive individuals. Additional spectral analysis of PORH showed that MST induced modest increase in cardiac and respiratory component of PORH response, rather than affecting vascular endothelium (Table 5). This was confirmed by unchanged microvascular AChID following MST at HS condition (Figure 3B). Taken together, these pivotal results indicate that changes in ANS due to HS diet do not directly contribute to microvascular endothelial dysfunction in normotensive individuals. In our earlier study we have demonstrated that 7-day HS diet is not accompanied by significant elevations of plasma volume [15], and present study clearly indicates that increase in plasma sodium due to 7-day HS loading leads to SNS suppression, but not sympathoexcitation. Thus, neither commonly accepted physiological compensatory mechanisms take place in the ANS regulation during 7-day HS loading in healthy normotensive individuals (such as cardiovascular regulation reflex activated by cardiopulmonary receptors through an increase in plasma volume [49], or both central and peripheral sympathoexcitation due to elevated plasma or cerebrospinal fluid NaCl concentration [50]) nor they have direct detrimental effect on microvascular reactivity. Still, observed findings that 24-h urine adrenaline/metanephrine level, along with the salt intake, could predict (negatively associated) microvascular AChID, together with the differences in HRV responses to MST at LS vs. HS diet suggest that in individuals who response to HS diet by sympathetic hyperactivity and/or stronger withdrawal of parasympathetic tone (especially under the influence of stress, i.e., who are salt-sensitive), changes in the ANS activity may possibly worsen the vascular impairment during HS diet. Yet, this potential relationship needs to be examined.

## 5. Conclusions

In conclusion, the present study has demonstrated that a 7-day HS diet beside affecting microvascular endothelium-dependent vasodilation independently of BP changes, also suppresses the SNS activity in young healthy normotensive individuals (i.e., salt-resistant), whereas direct correlation between microvascular endothelial function and changes in the ANS activity was not observed. Moreover, functional vascular alterations were not significantly affected by stress-induced increase in the SNS activity during HS intake. Results indicate that suppression of SNS during HS loading represents a physiological compensatory cardiovascular response, rather than direct pathophysiological mechanism by which short-term HS diet affect microvascular function in young normotensive individuals. Still, the switch from SNS suppression (representing normal compensatory response) to SNS activation (representing abnormal response) in condition of HS loading could be a breaking point which distinguish the salt-sensitive and salt-resistant cardiovascular response to dietary salt intake.

## Figures and Tables

**Table 1:** Anthropometric, Hemodynamic and Biochemical Responses to a 7-Day High-Salt Diet in Young Healthy Individuals.

Parameter	LS	HS
*N* (W/M)	47 (19/28)	
Age (years)	21 ± 2	
**Anthropometric Parameters**		
BMI (kg/m^2^)	23.9 ± 3.1	24.2 ± 3.2
WHR	0.82 ± 0.06	0.82 ± 0.05
**Hemodynamic Parameters**		
SBP (mmHg)	116 ± 12	117 ± 14
DBP (mmHg)	73 ± 9	72 ± 8
MAP (mmHg)	87 ± 8	87 ± 8
HR (beats per minute)	82 ± 15	79 ± 13
**Serum Biochemical Parameters**		
urea (mmol/L)	4.7 ± 1.3	4.7 ± 1.0
creatinine (µmol/L)	82 ± 14	77 ± 14
sodium (mmol/L)	138.4 ± 2.1	140.2 ± 2.5 *
potassium (mmol/L)	4.0 ± 0.2	4.2 ± 0.3 *
calcium (mmol/L)	2.47 ± 0.07	2.42 ± 0.09 *
iron (μmol/L)	20.2 ± 7.5	19.2 ± 6.4
transferrin (g/L)	2.71 ± 0.32	2.59 ± 0.31 *
glucose (mmol/L)	4.9 ± 0.5	4.9 ± 0.7
hsCRP (mg/L)	1.2 ± 2.4	0.9 ± 1.0
**24-h Urine Biochemical Parameters**		
24h urine volume (mL)	1618 ± 654	1642 ± 657
24h creatinine coefficient (µmol/24 h/kg)	180.5 ± 66.3	177.0 ± 47.0
24 h urine urea (mmol/dU)	318.5 ± 196.2	293.0 ± 90.1
24 h urine protein (mg/dU)	98.9 ± 53.1	100.6 ± 42.6
24 h urine albumin (mg/dU)	10.4 ± 17.5	7.0 ±4.9
24 h sodium (mmol/dU)	109.1 ± 51.6	247.6 ± 107.0 *
24 h potassium (mmol/dU)	46.0 ± 18.5	51.9 ± 19.9
calculated salt intake (g/day)	6.4 ± 3.0	14.5 ± 6.3 *

Data are presented as mean ± standard deviation (SD). LS—low salt; HS—high-salt; *N*—number of participants; W—women; M—men; BMI—body mass index; WHR—waist-to-hip ratio; SBP—systolic blood pressure; DBP—diastolic blood pressure; MAP—mean arterial pressure; HR—heart rate; hsCRP—high-sensitivity C reactive protein. * *p* < 0.05 LS vs. HS (Paired *t*-test).

**Table 2:** The Effect of a 7-Day High-Salt Diet on 24-h Urine Catecholamine and Catecholamine Metabolites Excretion in Young Healthy Individuals.

	LS	HS
*N* (W/M)	23 (7/16)	
Dopamine, µmol/dU	1.47 ± 0.050	1.40 ± 0.70
Noradrenaline, µmol/dU	0.71 ± 2.46	0.18 ± 0.08 *
Adrenaline, µmol/dU	0.03 ± 0.02	0.02 ± 0.02
Homovanillic acid, µmol/dU	23.25 ± 7.95	21.08 ± 8.17
Normetanephrine, µmol/dU	1.05 ± 0.34	0.93 ± 0.34 *
Metanephrine, µmol/dU	0.54 ± 0.16	0.45 ± 0.17 *
Vanillylmandelic acid, µmol/dU	16.98 ± 5.80	15.68 ± 6.50

Data are presented as mean ± standard deviation (SD). LS—low salt; HS—high-salt; *N*—number of participants; W—women; M—men. * *p* < 0.05 LS vs. HS (Paired *t*-test).

**Table 3:** The Effect of a 7-Day High-Salt Diet on 5-min Heart Rate Variability (HRV) in Young Healthy Individuals.

Parameter	LS	HS
*N* (W/M)	23 (7/16)	
Time Domain Analysis		
Mean HR (bpm)	68 ± 10	69 ± 9
Mean RR (ms)	902 ± 119	875 ± 103
SDNN (ms)	92 ± 32	79 ± 38
RMS-SD (ms)	82 ± 35	73 ± 43
pNN50 (%)	38 ± 18	35 ± 20
Frequency Domain Analysis		
TP (ms^2^/Hz)	2755 ± 1995	1848 ± 1580
VLF (ms^2^/Hz)	843 ± 557	774 ± 669
LF (ms^2^/HZ)	1205 ± 1051	687 ± 666 *
HF (ms^2^/Hz)	708 ± 683	598 ± 677
LF/HF	2.19 ± 1.40	1.77 ± 0.92

Data are presented as mean ± standard deviation (SD). HS—high salt; LS—low-salt; HR—heart rate; RR—RR interval; SDNN—Standard deviation of all NN intervals; RMS-SD—the square root of the mean of the sum of the squares of differences between adjacent NN intervals; pNN50%—NN50 count divided by the total number of all NN intervals; TP—total power; VLF—power in very low frequency range; LF—power in low frequency range; HF—power in high frequency range. * *p* < 0.05 LS vs. HS (Paired *t*-test).

**Table 4:** The Effect of Mental Stress Test (MST) on 5-min Heart Rate Variability (HRV) at Low-Salt (LS) and High-Salt (HS) Diet Conditions in Young Healthy Individuals.

Parameter	LS	LS+MST	LS	HS+MST
*N* (W/M)	24 (12/12)			
Time Domain Analysis				
Mean HR (bpm)	73 ± 12	72 ± 10	75 ± 9	71 ± 10
Mean RR (ms)	844 ± 144	844 ± 119	813 ± 98	854 ± 114
SDNN (ms)	84 ± 43	79 ± 35	84 ± 43	93 ± 50
RMS-SD (ms)	82 ± 58	71 ± 44	76 ± 60	89 ± 68
pNN50 (%)	35 ± 24	34 ± 21	32 ± 25	37 ± 25
Frequency Domain Analysis				
TP (ms^2^/Hz)	2905 ± 3593	2111 ± 1954	2399 ± 2504	3314 ± 3842
VLF (ms^2^/Hz)	803 ± 726	716 ± 537	768 ± 715	908 ± 753
LF (ms^2^/HZ)	1086 ± 1331	888 ± 1121	813 ± 922	1344 ± 1801
HF (ms^2^/Hz)	1016 ± 1659	507 ± 400	818 ± 1172	1062 ± 1586
LF/HF	1.84 ± 1.38	2.72 ± 3.20	1.74 ± 1.02	1.88 ± 1.27

Data are presented as mean ± standard deviation (SD). HS—high salt; LS—low-salt; MST—mental stress test; *N*—number of participants; W—women; M—men; HR—heart rate; RR—RR interval; SDNN—Standard deviation of all NN intervals; RMS-SD—the square root of the mean of the sum of the squares of differences between adjacent NN intervals; pNN50%—NN50 count divided by the total number of all NN intervals; TP—total power; VLF—power in very low frequency range; LF—power in low frequency range; HF—power in high frequency range.

**Table 5:** The Effect of Mental Stress Test (MST) on FFT Power Spectrum PORH LDF Signal at Low-Salt (LS) and High-Salt (HS) Diet Conditions in Young Healthy Individuals.

Parameter	LS	LS+MST	HS	HS+MST
*N* (W/M)	24 (12/12)			
**% of total power within frequency band**				
Cardiac (0.6–2.0 Hz)	6.39 ± 5.51	11.75 ± 11.46	5.58 ± 5.07	9.19 ± 9.58
Respiratory (0.15–0.6 Hz)	3.47 ± 3.28	6.84 ± 7.52	3.10 ± 3.25	5.86 ± 5.94
Myogenic (0.05–0.15 Hz)	3.65 ± 2.71	4.95 ± 4.95	3.09 ± 2.28	4.25 ± 3.43
Sympathetic (0.02–0.05 Hz)	14.40 ± 3.81	14.31 ± 4.60	14.59 ± 4.09	13.48 ± 4.40
Endothelial (0.008–0.02 Hz)	72.49 ± 9.99	61.92 ± 18.78	73.63 ± 9.66	67.86 ± 13.80

Data are presented as mean ± standard deviation (SD). FFT—Fast Fourier Transform; PORH—post-occlusive reactive hyperemia; LDF—laser Doppler flowmetry; HS—high salt; LS—low-salt; MST—mental stress test; *N*—number of participants; W—women; M—men. Statistical analysis: One-Way ANOVA Repeated Measure Test.

## Data Availability

The data presented in this study are available on request from the corresponding author.
